# The Hepatic Sinusoid in Chronic Liver Disease: The Optimal Milieu for Cancer

**DOI:** 10.3390/cancers13225719

**Published:** 2021-11-15

**Authors:** Albert Gibert-Ramos, David Sanfeliu-Redondo, Peio Aristu-Zabalza, Ana Martínez-Alcocer, Jordi Gracia-Sancho, Sergi Guixé-Muntet, Anabel Fernández-Iglesias

**Affiliations:** 1 Liver Vascular Biology Research Group, Barcelona Hepatic Hemodynamic Unit, Institut d’Investigacions Biomèdiques August Pi i Sunyer (IDIBAPS), 08036 Barcelona, Spain; algibert@clinic.cat (A.G.-R.); dsanfeliu@clinic.cat (D.S.-R.); anmartineza@clinic.cat (A.M.-A.); jordi.gracia@idibaps.org (J.G.-S.); 2 Barcelona Liver Bioservices, 08036 Barcelona, Spain; peio@liver.barcelona; 3 Centro de Investigación Biomédica en Red de Enfermedades Hepáticas y Digestivas (CIBEREHD), 28029 Madrid, Spain; 4 Hepatology, Department of Biomedical Research, Inselspital & University of Bern, 3008 Bern, Switzerland

**Keywords:** hepatocellular carcinoma, CLD, cirrhosis, LSEC, HSC, Kupffer cell, NASH, portal hypertension

## Abstract

**Simple Summary:**

During the development of chronic liver disease, the hepatic sinusoid undergoes major changes that further compromise the hepatic function, inducing persistent inflammation and the formation of scar tissue, together with alterations in liver hemodynamics. This diseased background may induce the formation and development of hepatocellular carcinoma (HCC), which is the most common form of primary liver cancer and a major cause of mortality. In this review, we describe the ways in which the dysregulation of hepatic sinusoidal cells—including liver sinusoidal cells, Kupffer cells, and hepatic stellate cells—may have an important role in the development of HCC. Our review summarizes all of the known sinusoidal processes in both health and disease, and possible treatments focusing on the dysregulation of the sinusoid; finally, we discuss how some of these alterations occurring during chronic injury are shared with the pathology of HCC and may contribute to its development.

**Abstract:**

The liver sinusoids are a unique type of microvascular beds. The specialized phenotype of sinusoidal cells is essential for their communication, and for the function of all hepatic cell types, including hepatocytes. Liver sinusoidal endothelial cells (LSECs) conform the inner layer of the sinusoids, which is permeable due to the fenestrae across the cytoplasm; hepatic stellate cells (HSCs) surround LSECs, regulate the vascular tone, and synthetize the extracellular matrix, and Kupffer cells (KCs) are the liver-resident macrophages. Upon injury, the harmonic equilibrium in sinusoidal communication is disrupted, leading to phenotypic alterations that may affect the function of the whole liver if the damage persists. Understanding how the specialized sinusoidal cells work in coordination with each other in healthy livers and chronic liver disease is of the utmost importance for the discovery of new therapeutic targets and the design of novel pharmacological strategies. In this manuscript, we summarize the current knowledge on the role of sinusoidal cells and their communication both in health and chronic liver diseases, and their potential pharmacologic modulation. Finally, we discuss how alterations occurring during chronic injury may contribute to the development of hepatocellular carcinoma, which is usually developed in the background of chronic liver disease.

## 1. Introduction

**Figure 1:**
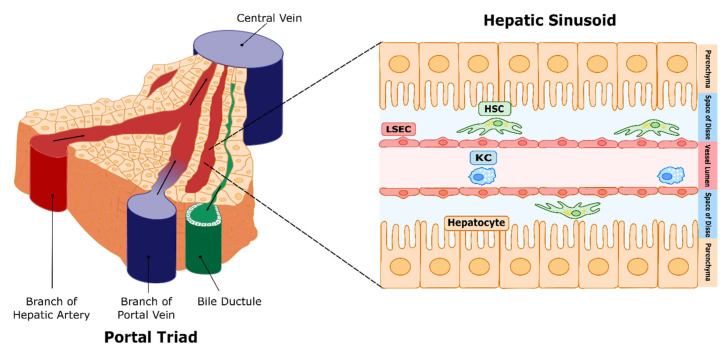
Hepatic circulation and microcirculation. Representation of the liver circulation, with the portal triad, which includes the hepatic artery, which supplies oxygenated blood; the portal vein, carrying blood rich in nutrients from the small intestine; and the bile duct, which collects bile products secreted by hepatocytes. Blood then mixes along the sinusoids, which are the liver microvessels (right panel), and drains into the central vein, which leads to the vena cava. Liver sinusoidal endothelial cells (LSECs) constitute the walls of the microvessel. Hepatic stellate cells (HSCs) reside in the space defined between LSECs and hepatocytes (space of Disse), and act as the sinusoidal pericytes, while Kupffer cells (KCs) (resident macrophages) are located in the sinusoidal lumen.

The liver is the main organ regulating blood clearance and metabolism, and as the first organ receiving intestinal blood, it participates majorly in the immune response. The hepatic sinusoid constitutes the microcirculatory bed of the liver, and it is highly specialised to facilitate all liver functions. As opposed to most other tissues in the body, the liver receives venous blood as an input, arriving through the portal vein, rich in nutrients and carrying approximately 50% of the hepatic oxygen supply. This blood mixes with the oxygenated arterial blood arriving from the hepatic artery and flows through the sinusoid, draining into the vena cava [1] (Figure 1). Due to this and other functional features, the hepatic microcirculation requires highly specialized cells which are different from those found in other vascular beds. The hepatic sinusoid is mainly composed of liver sinusoidal endothelial cells (LSECs), which constitute the permeabilized wall of the sinusoid, hepatic stellate cells (HSCs), which are vitamin A-storing pericytes localized in the space of Disse—or the perisinusoidal space, which is the area between hepatocytes and LSECs—and regulate the sinusoidal microvascular tone and synthesize extracellular matrix (ECM), and Kupffer cells (KCs), the liver-resident macrophages. The characteristic phenotype of the sinusoidal cell types is essential for hepatocyte function, and determines the physiology and pathology of the liver [2].

Under persistent damage, such as chronic hepatitis B or C, chronic alcohol and/or high fat diet consumption, among others [3], the specialized phenotype of all hepatic cell types is impaired. This induces persistent inflammation and wound healing mechanisms which, over time, will trigger the formation of scar tissue and hemodynamic alterations in the liver [4], leading to cirrhosis and hepatocellular carcinoma (HCC), which is currently the eleventh most common cause of death globally [5]. Concretely, at advanced stages of chronic liver diseases (CLD), the risk of HCC development is raised significantly, with around 80–90% of HCC appearing in a cirrhotic background [5,6].

Structural and dynamic alterations in the cirrhotic liver (fibrosis and microvascular dysfunction, respectively) lead to increased hepatic vascular resistance (HVR) to blood flow, causing an increase in portal pressure known as portal hypertension (PH). PH is the main complication of cirrhosis, and the cause of further complications such as the formation of portosystemic collateral circulation, gastroesophageal varices and bleeding, hyperdynamic circulation, ascites and hepatic encephalopathy, among others [7,8,9].

Therefore, CLD and the rise in HVR are dynamic and multifactorial processes characterized by the alteration of several pathways and cellular functions, involving all of the different hepatic cells (mainly LSECs, HSCs, KCs, and hepatocytes) during CLD progression and regression. Hence, a wide and deep understanding of these molecular mechanisms has been pursued in recent years in order to develop effective strategies and ameliorate PH by targeting its primary cause: altered liver microvascular circulation.

## 2. Cells from the Hepatic Sinusoid

### 2.1. Liver Sinusoidal Endothelial Cells

LSECs are highly specialized endothelial cells with a unique morphology and function. Like any microvascular endothelium, these cells constitute the interphase between blood and parenchymal cells (the hepatocytes), which carry out the main metabolic functions of the liver. However, LSECs differ from generic endothelial cells mainly by displaying multiple pores, or fenestrations, which are clustered together in groups known as “sieve plates” [2], and by the lack of an organized basement membrane, which makes them the most permeable barrier of the mammalian body [10,11]. These specialized features facilitate the diffusion of substrates between blood and the space of Disse, allowing direct exchange with hepatocytes and HSCs [12]. LSECs not only constitute a physical barrier but also have an active role in different physiological or pathological processes such as the modulation of the hepatic vascular tone, scavenging, metabolism, the immune response, and the driving of liver regeneration [13,14,15].

#### 2.1.1. LSEC Functions

##### Sieving Function

Fenestrae may have a diameter between 50 and 200 nm [16], and vary in size and number depending on their localization in the liver and on the species (e.g., they are larger in humans than in rodents) [17]. The periportal region presents larger but fewer fenestrae per sieve, while the centrilobular region fenestrae are smaller but more numerous [18], according to the intralobular oxygen gradient [19]. Fenestrae are dynamic, and change their diameter in response to different stimuli, including extracellular agents, liver diseases or ageing [20]. During CLD, LSECs lose fenestrae and become “capillarized”, being similar to an ordinary impermeable capillary. Therefore, LSEC capillarization is a common indicator of CLD, and it is hypothesized to be the first stage in liver fibrosis [21], contributing to further HSC and KC activation, among other processes [22,23,24]. The regulation of capillarization is not completely understood, although hedgehog ligands secreted paracrinally by HSCs and immune cells, as well as the DLL4 ligand, could be determinant factors in this process [25,26].

##### Modulation of Vascular Tone

Liver sinusoids are thought to be the main site of blood flow regulation within the liver. LSECs respond to changes in portal blood flow and pressure, and even produce vasoactive molecules to signal to HSCs and eventually regulate the sinusoidal diameter. Shear stress (frictional force applied by blood flow on the endothelial surface) is a main regulator of the vasoprotective transcription factor Krüppel-like factor 2 (KLF2), which induces the endothelial upregulation of vasodilating agents such as nitric oxide (NO) [27]. In the healthy liver, LSECs are the main source of NO [28] and maintain HSC quiescence through the equilibrium between the secretion of vasodilators and vasoconstrictors [29].

##### Endocytic Capacity

LSECs have been described as the endothelial cells with the highest endocytic capacity in the human body, performing a pivotal role in the clearance of blood-borne waste macromolecules and small particles through endocytic receptors [30]. Together with KCs, they belong to the reticuloendothelial system of the liver, where KCs are the professional phagocytes, being in charge of large particles, and LSECs are the professional pinocytes [31], contributing to around 45% of the total pinocytic vesicles of the rat liver. In order to fulfil their role as endocytic cells, LSECs display high-affinity endocytic receptors, including scavenger receptors, mannose receptor and Fc gamma-receptor IIb2 (CD32b), among others. These receptors, along with toll-like-receptors (TLR), participate in innate immunity as pattern recognition receptors (PRRs), which sense pathogen-associated molecular patterns (PAMPs) and damage-associated molecular patterns (DAMPs) secreted by apoptotic hepatocytes [14,32]. Finally, the endocytic capacity of LSECs has been described to include the clearance of blood-borne viruses and bacteriophages. In fact, hepatitis B virus, adenovirus 1, human immunodeficiency virus and T4 bacteriophages have been reported to be preferentially endocytosed by LSECs and not by KCs, as it was traditionally thought [33,34,35,36].

##### Immune Hepatic Tolerance

Due to its portal irrigation, the liver may be exposed to a variety of antigens arriving from the gut [37]. Therefore, the immune response in the liver should be able to discriminate between harmful antigens from pathogens and harmless ones from ingested food or common microbiota. This distinction (tolerance) is essential in order to avoid a permanent activation of immune responses in the liver which would damage the tissue [38]. In fact, the liver is known to favour tolerance rather than immunity [39]. The mechanisms by which this tolerance is achieved are still controversial, but several types of cells are thought to participate in hepatic tolerance, including LSECs [40].

LSECs are considered antigen-presenting cells (APCs), as they express major histocompatibility complexes I and II (MHC-I and MHC-II) [41,42]. However, when interacting with T cells, they do not induce an immunogenic response, but they enhance tolerance instead. Evidence suggests that when LSECs present an antigen to CD4+ T cells, they promote their differentiation towards the T regulatory (Treg) immunosuppressive cell type [43]. On the other hand, LSEC antigen presentation to CD8+ T cells increases programmed death in CD8+ T cells [44], therefore suppressing the immune response.

#### 2.1.2. LSEC Capillarization in Liver Injury

At early stages of liver damage, the alteration of blood shear stress leads to a profound downregulation of KLF2 in LSECs, and consequently to a downregulation of its vasoprotective target genes, followed by HSC activation and fibrosis development [27]. However, although flow is typically disturbed in advanced CLD, KLF2 is overexpressed in cirrhotic livers, probably due to a deficient compensatory mechanism being unable to maintain NO synthesis [27].

Recent studies have shown that during early cellular stress or liver injury, autophagy is activated in LSECs, which helps to maintain the normal sinusoid architecture and phenotype [45,46]. However, if stress or injury becomes chronic, the autophagic process is impaired, leading to LSEC dysfunction accompanied by reactive oxygen species (ROS) accumulation, the downregulation of the antioxidant response, the activation of HSCs, and a decrease in intrahepatic NO levels [46]. On the other hand, endothelial autophagy could have a detrimental role during LSEC capillarization, as it would induce the degradation of caveolin-1, an important protein involved in fenestration biology [47,48].

In addition, LSEC capillarization directly contributes to increased HVR by the enhanced activation of the COX-1-TXA2 vasoconstrictor pathway [49,50].

Aside from microvascular dysfunction, PH may be aggravated by other complications, such as sinusoidal thrombosis [51]. LSECs have been reported to respond to the mechanical stretch caused by the increased blood pressure in the portal vein through the transmembrane mechanosensing integrins [52]. This interaction activates the Notch1 receptor, which promotes the secretion of CXCL1. This chemokine attracts neutrophils to the sinusoids, which interact with platelets found in the bloodstream, inducing thrombosis, which further promotes PH [52].

On the other hand, during liver damage, LSECs further contribute to hepatic inflammation through the recruitment of immune cells [53] such as natural killer T cells (NKT) [54] and B lymphocytes [55]. In particular, during CLD, LSECs are influenced by DAMPs and pro-inflammatory mediators secreted by KC, activating the transcription of several adhesion molecules, such as ICAM, VCAM and stabilins, among others, which induce the adhesion and migration of leukocytes from the lumen of sinusoids into the liver tissue [56,57]. Furthermore, their antigen-presenting ability during CLD induces a switch in T cells, promoting their activation and a proinflammatory phenotype, rather than tolerance [58].

Importantly, LSECs’ close communication with the other hepatic sinusoidal cells promotes their deregulation and the development of a proinflammatory and profibrogenic microenvironment, further aggravating liver disease.

### 2.2. Hepatic Stellate Cells

HSCs represent ~10% of the resident liver cells, and they have numerous and important functions in hepatic biology. Traditionally, HSCs have been known for being the principal cell type responsible for the formation of fibrotic scars in liver disease under persistent injury. However, in the healthy liver, HSCs are mainly found in a quiescent state (qHSCs), in which their main functions are the storage and metabolism of retinoids and the regulation of vascular tone in response to vasoactive mediators [59,60].

#### 2.2.1. HSC Functions

##### Vitamin A Storage and Metabolism

HSCs are the main deposit of vitamin A in the whole human body [60]. Vitamin A (mainly retinol and its derivates) is an essential nutrient which plays important roles in embryogenesis, vision, immunity, reproduction and the maintenance of differentiated epithelial tissues [61,62].

Dietary vitamin A is intestinally absorbed in the form of chylomicrons [62], and is taken up by the liver [63], where the 80% is stored in HSCs as retinyl esters inside cytoplasmatic lipid droplets [64]. Moreover, HSCs express enzymes which participate in vitamin A synthesis, such as alcohol dehydrogenase and aldehyde dehydrogenase [65].

It is well known that lipid droplets are lost during the process of HSC activation [66,67]. However, vitamin A may not only represent a marker of quiescence but also indeed prevent HSC activation. In this regard, primary in vitro activated HSCs showed an amelioration in their phenotype when treated with vitamin A [68]. Therefore, additional studies have focused on the formation and biogenesis of these lipid droplets. Lin et al. [69] demonstrated that the protein perilipin 5 plays an important role in the formation of HSC lipid droplets, and in the maintenance of HSC quiescence. On the other hand, stored vitamin A can be mobilized when necessary to fulfil the requirements of the organism by mechanisms which are poorly understood [62]. One of the proposed mechanisms for vitamin A mobilization may be patatin-like phospholipase domain-containing 3 (PNPLA3), which is highly expressed in HSCs, and is upregulated in response to retinol deficiency [70].

##### Immunoregulation

HSCs can respond to certain antigens, such as lipopolysaccharide (LPS), and other bacterial products that activate the secretion of chemokines and cytokines that modulate the immune response [71,72,73]. Although less investigated, HSCs also express APC-related molecules such as MHC and CD80, which are necessary for T cell activation. However, the high production of cytokines by T cells promotes the activation of HSCs [71].

##### HSC Activation in Liver Injury

The activation of HSCs is a fundamental factor in CLD progression [74]. During liver injury, qHSCs are gradually activated, obtaining a more proliferative, migrating and contractile phenotype that increases the production of extracellular matrix molecules, leading to the fibrosis of the hepatic tissue and the contraction of the sinusoids [75].

HSC activation is triggered by different conditions, such as inflammation or interactions with other cell types and signalling pathways [75]. Damaged LSECs or apoptotic hepatocytes release several inflammatory molecules, such as TNFα, IL-6 or Hh ligands, and DAMPS that trigger an inflammatory response that stimulates HSC activation [75]. Moreover, vasoconstrictors released by capillarized LSECs further promote HSC activation. Indeed, the HSC phenotype is closely linked to LSECs’, as the restoration of the LSEC phenotype and functions (including the upregulation of KLF2 and increased NO levels) promotes HSC deactivation [76].

On the other hand, during hepatic fibrosis and inflammation, both the increased deposition of ECM proteins and the shift in its composition signal through the integrin pathway in HSCs, inducing their activation [77,78]. Proteoglycans such as kazal-like domain proteoglycan 1 (SPOCK1) are one example of extracellular matrix molecules found in human and rat fibrotic livers described to promote HSC activation through the integrin α5β1/PI3K/Akt signalling pathway [77].

High-energy metabolites are also an important factor in HSC activation and CLD progression. Indeed, lipid droplets in HSCs undergo beta-oxidation in order to provide energy for HSC activation, as demonstrated by the external oleic acid administration in vitro [67,79]. Furthermore, in the pathogenesis of non-alcoholic fatty liver diseases (NAFLD), leptin plays a key role in obesity development, the levels of which are usually elevated in the plasma of obese individuals [80], inducing HSC activation and promoting NAFLD development [81]. In this regard, leptin has been shown to directly activate HSCs through Hh signalling [82], and indirectly through the secretion of TGF-β1 by leptin-activated KCs [83].

The immune system is also involved in HSC activation, as a response to the presence of pathogens or endotoxins from Gram-negative bacteria, mainly LPS, inducing hepatic inflammation and fibrosis that can develop or further aggravate CLD [84,85]. Among other immune cells, NKT cells play an important role in the activation of HSCs during non-alcoholic steatohepatitis (NASH) progression [86,87], particularly through fibrogenic responses in HSCs by osteopontin and Hh ligand signalling [87]. On the other hand, Th17 lymphocytes and neutrophils contribute to HSC activation [88,89,90] through the secretion of IL-17 by a pro-inflammatory feedback loop: activated HSCs induce the Th17 cell response stimulating IL-17 production that activates HSCs [91]. As observed, HSCs are closely involved in the immune response, suggesting a key role during hepatitis C virus (HCV) infection. HCV particularly targets hepatocytes to replicate their RNA, which causes liver injury and apoptotic bodies that will trigger HSC activation and its profibrogenic phenotype [92]. However, studies have also shown that HCV can directly interact with HSCs, also inducing their activation [93,94,95].

On the other hand, different clinical results have demonstrated a relationship between liver fibrosis and HCC development [96]. Therefore, considering that HSCs are the main cell type responsible for ECM deposition and liver fibrosis, this suggests that HSCs may play an important role in tumour formation and development. For instance, activated HSCs promote HCC progression through the secretion of a large panel of cytokines, depending on the aetiology of the liver fibrosis [97].

### 2.3. Kupffer Cells

KCs are self-maintaining and non-migratory liver-resident macrophages which can be found within the lumen of hepatic sinusoids. KCs represent ~10% of hepatic cells and 80–90% of the tissue macrophages in the body. They participate in hepatic immune tolerance and danger sensing for the preservation of hepatic homeostasis. Importantly, KCs should not be mistaken for monocyte-derived macrophages, which are recruited to the liver only upon inflammation.

#### 2.3.1. Kupffer Cells’ Functions

KCs play a fundamental role in the innate and adaptive immune response during liver diseases. In homeostatic conditions, KCs display an anti-inflammatory phenotype (traditionally known as an M2 phenotype). KCs are responsible for the phagocytosis of bacteria or particle-associated antigens [98], and for the uptake of soluble antigens through fluid endocytosis [99]. Moreover, KCs may also interact with neutrophils and present them with captured pathogens for their degradation [100].

Despite their phagocytic activity and their ability to act as APCs, in healthy conditions KCs trigger a tolerogenic response to T-cells while competing with other cells with stronger APC activity, resulting in a low inflammatory response [101]. Healthy KCs also participate in haemoglobin degradation through its incorporation via the scavenger receptor CD163 and its degradation by heme-oxygenase I, resulting in vasoprotective products such as carbon monoxide [102].

Upon damage, KCs recognize PAMPs or DAMPS secreted by injured parenchymal cells and become proinflammatory, activating the inflammasome pathway and secreting proinflammatory cytokines, such as IL-1β and IL-18, as a defensive mechanism against pathogens [103,104,105]. This proinflammatory response may be relevant to keep potential infections under control and protect the liver against other forms of acute damage.

#### 2.3.2. Kupffer Cells in CLD

If KCs are chronically activated by continuous exposure to harmful substances or inflammatory factors, their persistent response may contribute to CLD progression [106,107,108]. Indeed, during CLD, chronic injury and associated PH may impair intestinal permeability and the composition of gut microbiota [109,110,111,112], which increases the risk of infections and may expose the liver to increased endotoxins and PAMPs. These would then be sensed by KCs through TLR and PRR, resulting in the persistent secretion of inflammatory cytokines (including IL-18, IL-12, IL-1β and TNFα) and the generation of oxidative stress, further contributing to inflammation [113,114,115,116].

In addition to pathogens, KCs may also be activated by the release of DAMPS or apoptotic bodies secreted by damaged hepatocytes, amplifying the inflammatory response by the recruitment of other immune cells such as neutrophils [117,118,119]. Moreover, apoptotic bodies engulfed by KCs induce their secretion of TNFα and death ligands, which further promote inflammation and the activation of HSCs, inducing fibrosis [120,121,122].

On the other hand, the persistent activation of hepatic macrophages may result in immune exhaustion, a scenario with inefficient and immunosuppressive macrophages which are unable to phagocyte pathogens, and which are associated with an increased risk of infections in patients [123]. Indeed, the blocking of PD-L1 (a marker of macrophage exhaustion) improved macrophage function in an animal model of chronic liver injury [123], suggesting that achieving an anti-inflammatory but also functional macrophage phenotype is of importance for CLD, and should be a matter of future study. For this reason, and considering their interactions with the other sinusoidal cells during liver injury, KCs are being studied as therapeutic targets for different liver diseases [107,108].

## 3. Cellular Communication in the Liver Sinusoid

As described above, non-parenchymal cells are strategically distributed in the hepatic sinusoid, an optimal environment for cell function and communication within the liver. This cellular crosstalk is essential for liver homeostasis, and is critical to preserve the normal phenotype of the cells, modulating their differentiation and activity [124].

**Figure 2:**
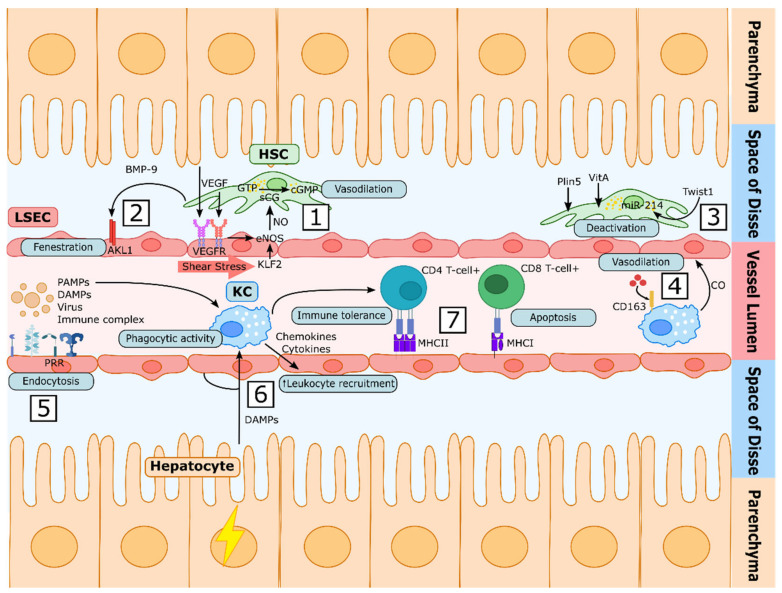
Liver sinusoidal cells’ communication under physiological conditions. (**1**) In homeostatic conditions, LSECs sense VEGF through specific receptors (VEGFR). In parallel, mechanical shear stress induces Krüppel-like factor 2 (KLF2), altogether maintaining LSECs’ vasodilatory phenotype, inducing NO synthesis. NO activates the soluble guanylate cyclase (sGC) in HSCs, leading to vasodilation. (**2**) The endothelium is fenestrated in healthy conditions. Circulating bone morphogenetic protein (BMP-9) released by HSCs contributes to the maintenance of the fenestrae by its recognition through activin receptor-like kinase 1 (ALK1). (**3**) Perilipin 5 (Plin5) participates in the formation of vitamin A (VitA)-containing lipid droplets. In addition, quiescent HSCs secrete exosomes containing the transcription factor Twist1, which promotes HSCs quiescence autocrinally through the transcription of miRNA-214. (**4**) Kupffer cells (KCs) incorporate haemoglobin through the scavenger receptor CD163 and, by its degradation, produce vasoprotective products such as carbon monoxide (CO). (**5**) LSECs display high-affinity receptors which participate in innate immunity, such as pattern recognition receptors (PRRs), being capable of sensing pathogen-associated molecular patterns (PAMPs), damage-associated molecular patterns (DAMPs), viruses and other immune complexes. (**6**) KCs are responsible for the phagocytosis of bacteria or particle-associated antigens. Upon damage, KCs recognize PAMPs or DAMPS and produce cytokines and chemokines which increase the expression of adhesion molecules by LSECs, leading to leukocyte infiltration and activation. (**7**) LSECs can act as antigen-presenting cells, as they express major histocompatibility complexes I and II (MHC-I and MHC-II). Presentation to CD4+ T cells promotes their differentiation towards T regulatory (Treg) immunosuppressive cells, enhancing tolerance. On the other hand, LSEC antigen presentation to CD8+ T cells increases the programmed death of the CD8+ T cells suppressing the immune response.

In homeostatic conditions, LSECs are constantly exposed to vascular endothelial growth factor (VEGF), which is derived from adjacent hepatocytes and stellate cells [21]. LSECs sense VEGF through VEGF receptors 1 and 2 (VEGFR1 and VEGFR2), which—together with laminar shear stress and low stiffness—maintains their vasodilatory phenotype, leading to a paracrine communication with HSCs, promoting their quiescence [78,125,126,127]. In this healthy scenario, LSEC–HSC crosstalk is mainly dependent on NO production by the endothelial NO synthase (eNOS). Indeed, NO released by LSECs activates the soluble guanylate cyclase (sGC)/cyclic guanosine monophosphate (cGMP)/protein kinase G pathway in HSCs [126], leading to myosin light chain relaxation (vasodilation). In addition, LSECs may release other vasodilator molecules (CO, prostacyclin) [49] or vasoconstrictors (TXA2, leukotrienes, endothelin-1) [50,128,129,130], maintaining a well-balanced equilibrium that allows the sinusoid to rapidly modulate its diameter in order to adapt to variations in intrahepatic blood pressure (Figure 2).

Despite VEGF being one of the most well-known endothelial regulators, other regulatory molecules have been described as being essential for the maintenance of a healthy LSECs phenotype [27,28]. The bone morphogenetic protein (BMP-9) is one example described recently; this circulating factor produced by HSC is recognized by its endothelial receptor activin receptor-like kinase 1 (ALK1), maintaining LSEC fenestrae and the expression of important differentiation markers [131].

Vasoprotective signals may not only be released in the form of soluble molecules, as described above; they may also be found in other forms, such as miRNAs or metabolites sometimes encapsulated inside extracellular vesicles such as microvesicles and exosomes [132,133,134,135]. In this regard, quiescent HSCs have been shown to release exosomes containing the transcription factor Twist1, which autocrinally stimulates the miRNA-214–connective tissue growth factor (CCN2) signalling pathway, overall maintaining their quiescent state [135].

Although HSCs are the last effectors of vasoconstriction and collagen synthesis, they may also have important effects in upstream events, modulating the phenotype of other hepatic cells. Indeed, directional cross-talk experiments demonstrated that the overexpression of the vasoprotective transcription factor KLF2 specifically in HSCs leads to the improvement of LSECs [136]. In this regard, recent transcriptomic studies suggest that HSCs represent relevant sources of cytokines that could paracrinally coordinate endothelial or immune cells and drive tissue repair [132].

### Sinusoidal Communication in CLD

Sinusoidal cells suffer a drastic transformation when they are exposed to damage and other environmental changes, which is thought to be the driving factor of fibrosis and other liver diseases [137]. Hence, the maintenance of a natural phenotype is crucial for the liver cells to carry out their functions.

Upon acute liver damage, sinusoidal communication is indispensable to trigger the orchestrated immunological response which is capable of rapidly controlling the inflammation process. Once hepatocytes detect the damage signals, they initiate the acute phase response, increasing their cytokine production, with IL-6 and IL-1 as primary cytokines. These molecules lead to a change in the phenotype of hepatocytes, leading to the major regulation of acute-phase protein production [138]. This, together with damage itself, leads to the release of a variety of DAMPS that are recognised by the activated neighbouring hepatocytes and non-parenchymal cells [139]. Moreover, hepatocytes may also increase the synthesis of VEGF during liver injury, inducing endothelial proliferation [140] or activating the release of growth factors—such as Wnt2 and hepatocyte growth factor (HGF)—that drive liver regeneration [141].

Pro-inflammatory KCs may also produce cytokines and chemokines which increase the expression of adhesion molecules by LSECs, leading to leukocyte infiltration and activation [57]. Leukocytes secrete pro-inflammatory mediators that activate HSCs which, in turn, release chemotactic factors that induce the transmigration and positioning of leukocytes [142]. If the injuring factor does not persist, the inflammatory process reaches the resolution phase, which is characterised by a switch towards a pro-resolution phenotype in macrophages, contributing to ECM degradation through the increased expression of matrix metalloproteinases. Additionally, during resolution, myofibroblasts may undergo apoptosis by expressing death receptors that can be recognised by NK cells [143].

**Figure 3:**
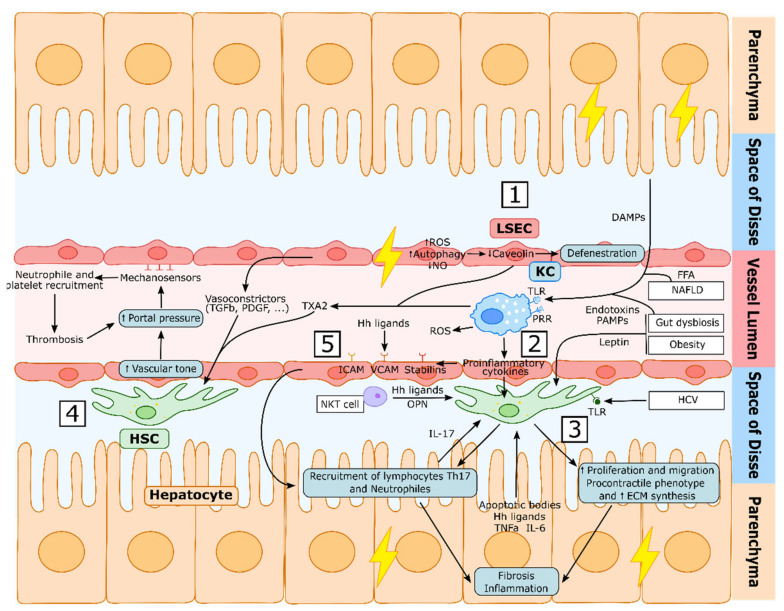
Liver Sinusoid dysfunction during chronic liver disease. (**1**) During chronic liver injury, LSEC become dysfunctional, impairing the autophagy process, increasing the generation of reactive oxygen species (ROS), decreasing nitric oxide (NO) intrahepatic levels and synthetizing increased vasoconstrictors, which induces the activation of HSCs. Hepatic damage further induces LSECs defenestration through the degradation of caveolin-1. (**2**) Kupffer cells (KCs) are activated by damage-associated molecular patterns (DAMPs), pathogen-associated molecular patterns (PAMPs), free-fatty acids (FFA) and endotoxins via their toll-like receptors (TLR) and pattern recognition receptors (PRR). This induces the secretion of reactive oxygen species (ROS) and proinflammatory cytokines that, together with other proinflammatory molecules secreted by other cell types, activate HSCs (**3**) which will acquire a proliferative, migrating, procontractile and proinflammatory phenotype that will induce liver fibrosis and inflammation. This procontractile phenotype increases vascular tone (**4**), which further increases portal pressure, activating LSECs mechanosensors that induce the recruitment of neutrophiles and platelets, the accumulation of which produces thrombi that will further increase portal pressure. LSECs are also activated by hepatocyte-derived hedgehog (Hh) ligands and other proinflammatory mediators (**5**) secreted during the inflammatory and injury process, which—via adhesion molecules such as ICAM, VCAM and Stabilins—will recruit leukocytes to the liver tissue, further promoting fibrosis and inflammation.

Altogether, sinusoidal communication in the liver is a highly regulated multidirectional process which may not only include paracrine signalling pathways between sinusoidal hepatic cells but also systemic stimuli or autocrine communication (Figure 3).

## 4. Therapeutic Approaches for Chronic Liver Disease

In the last few decades, the knowledge of the underlying mechanisms of the pathogenesis of cirrhosis has evolved notably. Although the prognosis of liver cirrhosis has improved [144], there are few pharmacologic strategies which achieve the regression of liver cirrhosis and its complications [145]. The current treatments mainly consist in non-selective betablockers (which mostly target extrahepatic complications of PH), surgical interventions (TIPS and transplantation), or the removal of the etiologic agent (diet, antivirals, alcohol abstention) [145,146]. Considering that CLD is a multifactorial disease, pharmacological therapies should be targeted to several key pathogenic targets and/or pathways [9,147]. In this section, we summarize the ongoing preclinical and clinical therapeutic options to improve CLD and its associated complications from a sinusoidal perspective (Table 1). Therapies for CLD or its complications from the physiological perspective are reviewed elsewhere [148,149,150,151].

### 4.1. Vasomodulation

As introduced above, during CLD the hepatic sinusoid becomes procontractile. LSECs synthesise increased vasoconstrictors while the expression of vasodilators is blunted. Therefore, different preclinical strategies are being evaluated in order to restore this balance and improve the dynamic component of intrahepatic vascular resistance.

#### 4.1.1. Inhibition of Vasoconstriction

COX-1 is one of the strongest vasoconstrictor signalling pathways known in hepatic microcirculatory dysfunction. This enzyme converts arachidonic acid into PGH2, which is further converted to TXA2, a vasoconstrictor that is over-synthesized by LSECs under chronic liver injury [49,50,210]. Our team demonstrated that cirrhotic livers treated with a selective inhibitor of COX-1 or with a TXA2 receptor blocker improved their microvascular dysfunction [152,153]. Similarly, Lin et al. also demonstrated that isolated LSECs from cirrhotic mice transfected with a COX-1 siRNA displayed downregulated TXA2 expression with subsequent liver fibrosis and portal pressure amelioration compared to the vehicle group [154]. Other prostanoid enzyme inhibitors/antagonists such as ifetroban [155] and nitroflurbiprofen [156] have been reported to alleviate liver fibrosis and inflammation, and to ameliorate NO bioavailability and PH in preclinical models of CLD.

Endothelin-1 is one of the most potent vasoconstrictor molecules involved in the physiological regulation of vascular tone. Several preclinical studies have demonstrated that the use of endothelin receptor antagonists improved the dysfunctional LSEC phenotype and liver fibrosis, and reduced portal pressure in preclinical models [157,158] and in patients with cirrhosis and PH [159,160].

#### 4.1.2. Induction of Vasodilation

Nitric oxide is the most potent vasodilator in the body. Therefore, several therapeutic approaches have targeted the NO pathway as a therapeutic option for PH [211,212]. One of these strategies is the modulation of sGC, the activity of which is dependent on NO, and which mediates the synthesis of cGMP in HSC, leading to relaxation. Indeed, the pharmacological modulation of the sGC is considered a better therapeutic option than NO donors [213], as direct NO administration may cause oxidative stress in the diseased liver. In this regard, preclinical studies with the sGC activators riociguat, praliciguat and BAY 60-2770 showed ameliorated PH, HSC deactivation, reduced fibrosis, improved intrahepatic vasodilation and vascular dysfunction, and reduced hepatic inflammation [127,162,163].

Phosphodiesterase-5 (PDE5) is the enzyme responsible for cGMP degradation. Therefore, PDE5-inhibitors (PDE5i) have been studied as an alternative to sGC activators in order to increase cGMP levels. Preclinical studies with the PDE5i sildenafil and udenafil reported increased NO-mediated vasodilation and improved endothelial dysfunction [164,165,166]. These therapeutics have been shown to prevent cGMP degradation in two clinical studies, leading to a reduction in portal pressure [167,168].

On the other hand, NO bioavailability may decrease in oxidative conditions as a result of its reaction with peroxide, resulting in peroxynitrite [214]. Therefore, antioxidant strategies have shown vasodilatory effects in the hepatic microcirculation of animal models of CLD, which may be due to a combination of both increased NO bioavailability and reduced cellular damage [169,170,201].

Statins are inhibitors of 3-hydroxy-3-methyl-glutaryl-coenzyme A reductase with lipid-lowering properties that are commonly advised for patients who are at risk of cardiovascular events [215]. For many years, preclinical and clinical studies have assessed the use of statins in CLD due to their pleiotropic effects in vascular diseases [215]. Indeed, their vasoprotective effects have been robustly demonstrated in compensated cirrhosis, reducing the risk of decompensation [216], and in decompensated cirrhosis, improving PH, intrahepatic vascular resistance (IHVR), and hepatic microvascular dysfunction [171,172]. Mechanistically, these drugs are the most potent pharmacologic activators of KLF2, which induces the synthesis of its vasoprotective target genes, promoting NO synthesis and HSC deactivation directly or indirectly through cellular crosstalk. In addition, the effects of statins have been validated in preclinical models of CLD with different aetiologies, including NASH, ameliorating liver fibrosis by enhancing NO bioavailability in LSECs, and consequently improving the HSC phenotype [45,76,136,173,174,175,176,177,178]. Similarly, these molecular effects of statins on aHSCs have also been validated on primary human aHSCs and on the LX-2 cell line [179].

Aside from their direct antifibrogenic effects, statins have also shown additional beneficial effects on other hepatic complications associated with CLD, such as in animal models of haemorrhage/resuscitation, infection, and acute on chronic liver failure. These effects would be mediated by the prevention of endothelial dysfunction and an associated increase in eNOS; a reduction in oxidative stress and inflammation; and improved liver hemodynamics and survival [180,181,182]. Although simvastatin is generally safe and its use is encouraged [217], recent preclinical studies were aimed at developing statin-loaded, liver-targeted polymeric micelles as an alternative approach in order to reduce cytotoxicity [218].

Farnesoid X receptor (FXR) is a transcription factor implicated in bile and lipid metabolism, with a high expression in the liver [219], which regulates a variety of vasoprotective enzymes. Obeticholic acid (OCA) is a potent and selective FXR agonist [220], the administration of which showed beneficial effects on PH by reducing IHVR in preclinical models of cirrhosis. Indeed, these studies suggested that OCA could directly target LSECs and KCs, inducing the elevated expression and activity of eNOS and decreased hepatic inflammation [184,185]. Currently, there are various RCTs evaluating OCA in patients with PH and CLD [221,222]. Specifically, one of these has just achieved phase 4, and is being tested on patients with primary biliary cholangitis (PBC), while another one is in phase 3, being tested on adults with compensated cirrhosis with NASH aetiology.

#### 4.1.3. Targeting other Vascular Alterations

Cirrhosis is also characterized by a procoagulant microenvironment [223] and altered angiogenesis [224]. Cirrhotic rats treated with the anticoagulants enoxaparin or rivaroxaban ameliorated their HSC phenotype, liver microthrombosis, hepatic fibrosis and PH [186,187]. Nevertheless, another preclinical study demonstrated no beneficial effects from enoxaparin treatment on PH [225]. These controversial results indicate that new studies are required in order to conclude whether the use of anticoagulants could be effective for PH.

Tyrosine kinase receptor inhibitors were suggested as a therapeutic option for angiogenesis occurring in advanced CLD [188,189]. In this context, rats with intrahepatic PH treated with sorafenib showed the inhibition of endothelial angiogenic and proliferative markers such as VEGFR-2 and PDGFRβ through the suppression of the Raf/MEK/ERK signalling pathway. Additionally, animals treated with sorafenib showed HSC phenotype amelioration, resulting in PP reduction [188]. In accordance with this, it has been demonstrated that sorafenib ameliorates the HSC phenotype by decreasing ECM deposition and the expression of fibrinogenic molecules in liver fibrosis [189].

### 4.2. Cell Death and Inflammation

The cell death that occurs during chronic injury may lead to further inflammation and liver damage. With this rationale, several anti-apoptotic approaches have been assessed in CLD. A pre-clinical study evaluating the pan-caspase inhibitor Emricasan demonstrated that the treated cirrhotic rats improved their LSEC and HSC phenotypes, resulting in the amelioration of hepatic microvascular dysfunction, with a marked reduction in liver fibrosis, PH and liver function [190]. However, these effects were not translated to patients with NASH cirrhosis and severe PH, which did not improve HVPG or other clinical parameters [191]. ASK1, an apical mitogen-activated protein kinase, has been implicated in apoptosis, inflammation, and fibrosis. Similarly to the case of Emricasan, the treatment of compensated NASH-cirrhotic patients with selonsertib, an ASK1 inhibitor, did not show any anti-fibrotic effects [192]. All of these clinical results suggest that the mechanisms affecting cell death may differ in humans and animal models, indicating that, in the former, cell death could occur through necroptosis or other caspase-independent pathways [226].

Peroxisome proliferator-activated receptors (PPARs) regulate the expression of the genes involved in lipid metabolism. However, these transcription factors participate in a wide variety of other molecular processes, including inflammation, insulin resistance, and fibrogenesis. There are three PPAR isoforms: PPAR-α, PPAR-δ (also known as PPAR-β), and PPAR-γ, all of which are expressed in the liver [227]. In hepatic sinusoids, PPAR-α is involved in the regulation of NO bioavailability, either by promoting its synthesis by LSECs or by preventing its scavenging by ROS [193]. Indeed, the activation of PPARs prevents the expression of cell adhesion molecules in LSECs, leading to the reduced recruitment of macrophages and determining their anti-inflammatory phenotype [194], altogether preventing the activation of HSC. Moreover, cirrhotic animals treated with the pan-PPAR (α/δ/γ) agonist lanifibranor ameliorated their PH mainly by improving their sinusoidal cell phenotype, leading to a reduction of microvascular dysfunction, fibrosis and inflammation [195]. Therefore, PPAR signaling may represent a therapeutic target for CLD, especially in the context of advanced CLD such as NASH and cirrhosis. Ongoing phase 3 RCT assessing lanifibranor and other PPAR agonists will elucidate the translatability of these drugs to the clinical practice [228,229,230,231].

Similarly, antidiabetic drugs have also shown antifibrotic effects, overall reducing portal pressure. Although their exact mechanisms of action remain unknown, it is hypothesized that their antifibrotic effects derive from an improvement in hepatic inflammation as a result of their action in metabolic pathways and insulin resistance. However, these effects would also be direct on HSCs, as the treatment of isolated HSCs in vitro with liragrlutide blunted their contractile activity, proliferation and profibrotic markers [196]. When administered to cirrhotic animals, they displayed reduced fibrosis, improved microvascular function and reduced portal pressure [196]. Liraglutide’s effects were further assessed in the LEAN phase 2 RCT in patients with NASH, leading to a significant reduction of fibrosis and a significant improvement in its histological resolution [197], while a recent RCT showed the resolution of NASH without an improvement in the fibrosis stage after treatment with semaglutide, another GLP-1 agonist, compared with the placebo group. [198]. Previous preclinical studies approaching other diabetes-related pathways with metformin and an anti-leptin receptor antibody point in the same direction, improving PH and HSC activation in cirrhotic rats [199,200].

### 4.3. Strategies Targeting Fibrogenesis

As explained above, liver architectural alterations are the result of fibrogenesis occurring during chronic liver injury and leading to PH. The LOX protein family participates in the cross-linking of collagen fibers, leading to fiber stabilization. In this regard, therapeutic strategies targeting LOX showed promising potential in preclinical models of fibrosis [232]. Unfortunately, trials assessing anti-LOX2 antibodies in patients with cirrhosis have been reported as negative [233,234].

### 4.4. Lifestyle and Dietary Interventions

#### 4.4.1. Microbiota

PH per se may induce severe changes in the gut, including gut dysbiosis and alterations in intestinal wall permeability. These alterations may expose the liver to infections and altered gut-derived factors, and may in turn activate the immune response [235], ultimately contributing to the development of CLD, independently of the aetiology [236,237,238]. For these reasons, the regulation of gut microbiota has been a focus of interest recently as a potential target for CLD therapy. The use of probiotics (live bacteria present in particular foods or ingredients) and prebiotics (which promote the growth and activity of the endogenous healthy microbiota) may have a protective role in hepatic hemodynamic [202,203,204,205], although there is controversy due to additional studies suggesting otherwise [206,207].

#### 4.4.2. Diet and Nutraceuticals

Nutraceuticals and natural bioactive compounds, which are molecules present in food that have a beneficial impact on biological processes at a physiological level [239], have also been studied as an alternative to pharmacological therapies due to their relative safety and accessibility. Lifestyle changes that include diet and physical exercise have been considered for the prevention and reduction of NAFLD progression [240,241]. Indeed, the Mediterranean diet has proven to be beneficial in NAFLD development, mainly due to its high content of polyphenols, vitamins and other molecules with anti-inflammatory and antioxidant effects [242,243]. Specifically, polyphenolic compounds such as resveratrol [244,245] or curcumin [208], or the omega-3 fatty acid, docosahexaenoic acid (DHA) [246], have also shown an improvement in different characteristics of CLD, mainly regarding oxidative stress, inflammation and lipid accumulation, accompanied by the amelioration of HSCs, hepatocytes and KC functions [247,248].

### 4.5. Sinusoidal Cell-Targeted Therapies

As described above, many of the preclinical findings end up having translatability issues when tested in clinical trials. These can be due to drug unspecificity for a certain cell type or selective cytotoxicity. Therefore, several studies have evaluated drug delivery strategies such as loaded nanoparticles or oligonucleotides that target specific cell types.

Indeed, He et al. designed and tested mannose-modified trimethyl chitosan-cysteine conjugate nanoparticles containing siRNA against TNFα which would target liver macrophages [249]. This treatment showed positive effects on the injured liver, reducing excessive inflammation and further liver damage. Similarly, it has been shown that nanoparticles coated with retinol are captured by HSCs, which, when loaded with antifibrotic molecules such as JQ1, atorvastatin [250] or NO improve the HSC phenotype, resulting in the improvement of liver fibrosis and PH [251].

On the other hand, therapies targeting mainly HSCs may also include the use of antisense oligonucleotides [252] and siRNAs-loaded lipoplexes [253] to ameliorate liver fibrosis and cirrhosis progression [254]. Altogether, the development of novel cell type-specific delivery systems may be a useful tool in order to target the liver sinusoids, and may become a second chance for drugs with promising results in pre-clinical studies but with undesired side effects and toxicity at the bedside.

## 5. CLD and the Sinusoidal Microenvironment in the Development of HCC

As mentioned above, cirrhosis is the main cause of HCC. HCC is the fourth cause of cancer death worldwide, with a survival rate of roughly 20% [255]. Resection, transplantation and ablation are the recommended treatments for the early stages of the disease. For cases in which surgery is not an option, the approved pharmacologic strategies include multi-kinase inhibitors, immunotherapy and antiangiogenics, or a combination of these. However, their prognostic is still poor, and may also be negatively affected by the stage of CLD [255].

Because CLD shares some alterations with HCC, it is conceivable that the CLD environment would represent a persistent source of pro-cancer stimuli for hepatocytes. Below, we will expose some hypotheses that could explain why and how CLD would induce or participate in the different stages of tumour development.

In general, the first stages of carcinogenesis start with DNA mutations. Indeed, healthy cells have complex DNA-repairing systems and checkpoints that prevent cells with defective DNA from proliferating, and instead induce their apoptosis. However, these tumour-suppressing mechanisms may still fail sometimes, and therefore cells under higher stress and DNA damage may have increased chances to become carcinogenic. In this regard, during CLD, the sinusoidal microenvironment rich in proinflammatory cytokines and ROS, alone or in combination with the source of liver injury (e.g., alcohol, a virus, excessive lipids) may induce increased DNA damage and promote de novo carcinogenesis [256,257]. Furthermore, the suppressed state of the immune system during CLD may contribute to the altered removal of dysfunctional cells [123,258,259,260], further enhancing tumour initiation.

In contrast to healthy hepatocytes, which have a proper oxygen supply (in accordance to the oxygen gradient along the hepatic sinusoids) [261], it is well accepted that hepatocytes in a cirrhotic liver may live in a hypoxic microenvironment due to the loss of fenestrae in the endothelial cells [262] (oxygen would not be able to traverse freely from the lumen to the space of Disse). This hypoxic condition could induce the death of normal hepatocytes, and would lead to the selection of those cells with a higher anaerobic metabolism. Because the tumour is usually characterized by an anaerobic environment due to the lack of vasculature, pre-selection by hypoxia may enhance the survival of HCC cells and tumour progression. On the other hand, other vascular deregulations in the cirrhotic liver, such as altered angiogenesis, might participate in tumour neovascularization, while cytokines and membrane proteins which are highly expressed (such as TGF-β2, PD-L1&2) [263] or downregulated (CD32b, Stab-2 and LYVE-1) [264] during CLD may inhibit T-cell antitumoral functions [44] and induce tumour cell proliferation and invasion.

**Figure 4:**
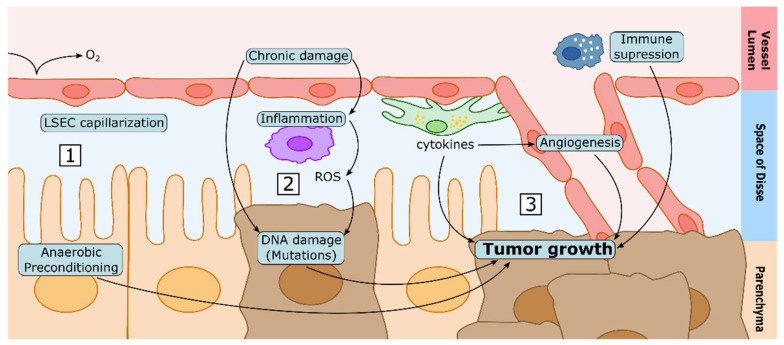
Proposed mechanisms linking CLD and HCC development. (**1**) The capillarization of LSECs may lead to less oxygen diffusing to the space of Disse. Therefore, hepatocytes may be preconditioned to anaerobic metabolism in CLD, which may represent an advantage to tumoral cells in hypoxic conditions. (**2**) Chronic damage and the associated chronic inflammation are known causes of DNA damage, which may lead to cellular dedifferentiation and tumorigenesis. (**3**) Cytokines released by the different hepatic cell types in conditions of chronic liver damage may induce tumour growth directly, enhancing neovascularization, or by suppressing the immune system.

Altogether, many of the sinusoidal alterations occurring in CLD described above may not only be detrimental for CLD itself; they may constitute a pro-HCC microenvironment to take into consideration in the pharmacological treatment of HCC, for instance by cotreatment strategies against HCC and CLD (Figure 4). However, further studies assessing the sinusoidal contribution to HCC in the context of CLD are still needed to validate these hypotheses.

## 6. Conclusions

The liver sinusoid is composed of highly specialized cells that maintain hepatocyte and liver function. However, persistent damage due to disease or exposure to toxic substances induces the deregulation of these cell types, which switch from maintaining proper liver homeostasis to a proinflammatory and profibrotic phenotype that further compromises liver function.

Because of the crucial role of sinusoidal cell types in the initiation and progression of CLD, the current research is focused on therapies that target these cells and their associated processes, such as the modulation of LSECs’ vasoactive capacities, or the pathways involved in cell death or inflammation. Some of these therapies have been shown to reduce fibrogenesis and portal hypertension, diminishing the strain on the liver and ameliorating its function in preclinical models that, combined with novel promising cell-targeted delivery strategies directly targeting sinusoidal cells, could avoid drug unspecificity or cytotoxicity.

On the other hand, HCC mostly develops in the CLD microenvironment [265]. Therefore, studies on HCC progression and regression, including drug development, should consider the role of the dysfunctional liver sinusoid during CLD. Future research should focus on a better understanding of the pivotal role of sinusoidal cells in driving liver disease and the development of HCC, not only to develop suitable and targeted therapies with low cytotoxicity and high efficacy but also to improve liver disease detection and prevention.

## Figures and Tables

**Table 1:** Therapeutic strategies with described effects improving the sinusoidal cell phenotype. We classified the reported effects of each study according to whether they directly targeted the main cells of the sinusoid (direct cellular effects), the hemodynamics of the liver (the dynamic component of hepatic vascular resistance), or structural effects (static component of hepatic vascular resistance, e.g., fibrosis or necrosis). ACLF, acute on chronic liver failure; ACLD, advanced chronic liver disease; ASK1, apoptosis signal-regulating kinase 1; BDL, common bile duct ligation; CH, cirrhosis; CTP, Child-Turcotte-Pugh; eNOS, endothelial nitric oxide synthase; ET, endothelin receptor; HFD, high-fat diet; HFGFD, high-fat glucose-fructose diet; HSC, hepatic stellate cell; HVPG, hepatic venous pressure gradient; HVR, hepatic vascular resistance; LSEC, liver sinusoidal endothelial cell; MAP, mean arterial pressure; MELD, model for end-stage liver disease; PH, portal hypertension; PP, portal pressure; PPP, portal perfusion pressure; sGC, soluble guanylate cyclase; SOD, superoxide dismutase; TAA, thioacetamide; TXA2, thromboxane A2; TXB2, thromboxane B2; TP, prostaglandin-endoperoxide.

Mechanism	Drug	Administration Method	Experimental Model	Structural Effects	Hemodynamic Effects	Direct Cellular Effects	Reference
Inhibition of vasoconstriction	SC-560 (COX-1 selective inhibitor) and SQ 29,548 (TXA2 receptor antagonist)	5 µM during 15 min before the hemodynamic study. Preincubation of the liver.	Male Wistar CCl_4_-cirrhotic rats	-	↑ Vasodilatory response to acetylcholine, ↓TXB2	-	[152]
	Terutroban (TP-receptor antagonist)	30 mg/kg once a day for 2 weeks after development of ascites	Male Wistar CCl_4_-cirrhotic rats	↓Fibrosis, ↓collagen I and TGF-β mRNA, ↓α-SMA protein	↓ PP, ↓ HVR	-	[153]
	COX-1 siRNA	Intravenous injections with 0.6 mg/kg every other day at 8 weeks after starting CCl_4_-cirrhosis induction	Male C57BL/6 CCl_4_-cirrhotic mice	-	↓ PP	Inhibition of the COX-1/TXA2 pathway	[154]
	Ifetroban sodium (TX receptor antagonist) or CGS 12970 (TX synthase inhibitor)	3 mg/kg or 10 mg/kg, respectively, every day, starting the last 2 weeks of alcohol treatment	Male Wistar alcohol and fat-induced cirrhotic rats	↓ Necrosis, ↓ inflammation and ↓ fibrosis ↓NF-kB activation, ↓TNFα, COX-2 and TGF-β1 expression.	-	-	[155]
	Nitroflurbiprofen or flurbiprofen	In vivo: 45 mg/kg or 30 mg/kg, respectively, intraperitoneally injected 24 h and 1 h prior to the hemodynamic and perfusion experiments. In vitro: From 25 to 250 umol/L	Male Wistar TAA-cirrhotic rats	-	↓ PP, ↓ splanchnic hyperemia, ↓HVR, ↑ vasodilation, ↓TXB2	In vitro: ↓ HSC contraction	[156]
	ET-A (ABT-627), ET-B (A-192621) or a mixed ET receptor antagonist (A-182086)	50 mg/kg, 40 mg/kg, or 30 mg/kg, respectively, once a day for 8 weeks during cirrhosis induction	Male BALB/c CCl_4_-cirrhotic mice	↓Fibrosis, ↓α-SMA protein and collagen I mRNA	↓ PP	-	[157]
	BQ-123 (ET-A antagonist)	10 nmol/min infused via a catheter in the mesenteric vein for 10 min	Healthy male Wistar rats	-	↓ PP	↑ Number and diameter of fenestrae	[158]
	BQ-123 (ET-A antagonist)	1000 and 3000 nmol/min infused for 20 min	16 CH patients with PH	-	↓ MAP and pulmonary vascular resistance index. No effects on HVPG.	-	[159]
	BQ-123 or Ambrisentan (ET-A antagonists)	300, 500, 1000 and 2000 nmol/L of BQ-123 infused through the hepatic artery. 5 or 10 mg single oral administration of ambrisentan	26 CH patients	-	BQ123: Vasodilation of the hepatic artery, ↓ HVPG. Ambrisentan: ↓ HVPG	-	[160]
	Ambrisentan (ET-A antagonist)	2 or 30 mg/kg/day for 2 weeks alone or in combination with 10 mg/kg/day of atorvastatin.	Male Sprague-Dawley rats with NASH induced by a HFGFD.	↓ NAS score	↓ PP, ↓ HVR	Improved markers of microvascular dysfunction, ↓ HSC contraction.	[161]
Induction of vasodilation	BAY 60-2770 (sGC activator)	0.3 mg/kg daily for 1 week	LSEC and HSC isolated from healthy and TAA-cirrhotic male Sprague-Dawley rats	↓ Fibrosis, ↓ Cirrhosis	-	Restoration of LSEC phenotype and quiescence of HSC	[127]
	Riociguat (sGC stimulator)	1 mg/kg daily, for 1 to 3 weeks	Male Sprague Dawley cBDL or CCl_4_-cirrhotic rats	↓ Fibrosis, ↓ Inflammation	↓ PP, ↑ vasodilation pathways	↓ HSC α-SMA expression	[162]
	Praliciguat (sGC stimulator)	STAM/HC: 3 or 10 mg/kg/day for 6 weeks during cirrhosis induction. TAA: 1, 3 or 10 mg/kg/day for 4 weeks during cirrhosis induction. CCl_4_: 1, 3 or 10 mg/kg/day for 6 weeks during cirrhosis induction.	Male C57/B6 mouse model with steatosis and metabolism with high cholesterol (STAM/HC), and TAA or CCl_4_-induced cirrhotic Sprague-Dawley rats	↓ Fibrosis, ↓ Inflammation	↓ MAP	↓ TGF-β-induced HSC activation, ↓TGF-β and PDGF-b, ↓ Macrophage infiltration	[163]
	Sildenafil (PDE5 inhibitor)	0.25 mg/kg twice a day for 1 week, starting 3 weeks after the surgery	Male Sprague-Dawley rats with cBDL-induced cirrhosis	↑ BH4, total hepatic biopterin and GTPCH-I activity	↑ sinusoid area, volumetric flow and perfused sinusoids ↓ PP, ↓ PPP	↑ NO bioavailability, ↑ phosphorilation of eNOS and Akt, ↑ NOx production	[164]
	Udenafil (PDE5 inhibitor)	Series A: 1, 5 or 25 mg/kg/day, starting 1 week after the cBDL surgery and continued for 3 weeks. Series B: Single dose of 5 or 25 mg/kg 4 weeks after surgery	Male Sprague-Dawley cBDL-cirrhotic rats	-	↓ PP	↓ HSC mRNA expression of procollagen type I and α-SMA	[165]
	Udenafil	1 mg/kg and 5 mg/kg for 60 min	Male Sprague Dawley cBDL or CCl_4_-cirrhotic rats	-	↓ PP, ↓ HVR, ↑ intrahepatic vasodilation	↑ eNOS protein, ↑ cGMP	[166]
	Vardenafil (PDE5 inhibitor)	A single dose of 10 mg	18 CH patients	-	↑ Portal blood flow, ↓ PP	-	[167]
	Udenafil	12.5, 25, 50, 75 and 100 mg daily for one week	35 patients with compensated liver cirrhosis and HVPG ≥12 mmHg	-	↓ HVPG, MAP	-	[168]
	Tempol (SOD mimetic)	In vivo: 180 µmol for 30 min during the hemodynamic study. In vitro: 50 µM for 6 h.	In vivo: Male Wistar CCl_4_ cirrhotic rats. In vitro: LSEC isolated from treated Wistar rats incubated for 6 h with a superoxide dismutase inhibitor.	In vivo: ↓ oxidative stress, ↑ cGMP.	↓ PP, ↑ portal blood flow, ↓ vascular resistance, ↓ MAP	In vitro: ↓ oxidative stress, ↑ NO.	[169]
	rMnSOD (recombinant manganese SOD)	Healthy rats: 15 µg/kg 2 h before the experiment. Cirrhotic rats: 15 µg/kg daily for 7 days. In vitro: 1 µM overnight	Male Wistar healty, CCl_4_ and cBDL-cirrhotic rats. In vitro: LX2 cells	↓ oxidative stress, ↓ deposition of fibrillar collagen	↓ PP, ↓ HVR, ↑ vasorelaxation	In vitro: ↓ oxidative stress, ↓ α-SMA and collagen I gene expression	[170]
	Simvastatin	One time dose of 40 mg, 30 and/or 60 min before the study	30 CH patients with HVPG ≥ 12 mm	-	↑ Hepatic blood flow, ↓ HVR	↑ NO levels	[171]
	Simvastatin	20 mg/day for 15 days, and 40 mg/day the following 15 days	59 patients with CH and PH	-	↓ HVPG, ↑ liver perfusion and function	-	[172]
	Atorvastatin, mevastatin, simvastatin or lovastatin	0.1, 1 or 10 µM for 24 h	LSEC isolated from male Wistar CCl_4_-cirrhotic rats	-	-	↑ KLF2, eNOS and thrombomodulin mRNA expression	[76]
	Simvastatin	LX-2 cells: 0.1, 1 and 10 µM for 24 and 72 h. HSC: 10 uM	LX-2 cells and primary HSC	-	-	↑ KLF2 mRNA expression, ↓ α-SMA mRNA and protein expression	[136]
	Atorvastatin	15 mg/kg once per day for 1 week	Male Sprague-Dawley BDL-cirrhotic rats	-	↓ PP, ↓ HVR, ↓ shunting	↑ eNOS mRNA, protein expression, ↑ PKG activity, ↓ HSC contraction	[173]
	Atorvastatin	15 mg/kg daily. Prophylaxis group: 1, 2, 3 or 5 weeks of treatment after BDL. Therapy group: 1 week of treatment at different time points after BDL.	Male Sprague-Dawley BDL-cirrhotic rats	Prophylaxis: ↓ fibrosis, Therapy: ↓ fibrosis, apoptosis.	-	Prophylaxis: ↓ ECM and HSC activation. Therapy: ↓ ECM, ↓ HSC activation, proliferation and apoptosis.	[174]
	Simvastatin	25 mg/kg/day for 3 days	Male Wistar CCl_4_-cirrhotic rats	↑ eNOS activity	-	↑ LSEC function	[175]
	Simvastatin	Chronic treatment: 20 mg/kg/day by gavage. Acute treatment: incubation of the portal-systemic collateral vascular bed for 25 min with 10 µM	Male Sprague-Dawley with portal hypertension induced by partial portal vein ligation	-	↓ PP, ↓ collateral vascular resistance	↑ SRS eNOS, COX-2 and TXA2 mRNA expression	[176]
	Simvastatin	20 mg/kg by gavage from 2 days prior to ligation until 7 days after the operation	Male Sprague-Dawley with portal hypertension induced by partial portal vein ligation	-	↓ PP, ↓ collateral vascular resistance	-	[177]
	Simvastatin, Atorvastatin	10 mg/kg/day of one drug for 2 weeks	Male Sprague-Dawley OFA rats with NASH induced by a HFGFD	↓ Steatosis, ballooning and inflammation, ↓ histological NASH	↓ PP	↑ eNOS and AKT phosphorylation, restoration of LSEC phenotype and quiescence of HSC	[178]
	Simvastatin	In vivo: 4 mg/kg/day for 8 weeks. In vitro: 10 µM for 24 h	In vivo: male Wistar rats with NASH induced by HFD. In vitro: LX-2 cell line activated with TGF-β.	In vivo:↓ liver inflammatory cells infiltration, ↓ steatosis, ↑ mRNA and protein eNOS ↓ iNOS and collagen I.	-	In vivo: ↓ liver inflammatory cells infiltration. In vitro: ↓ LX-2 activation, ↓ mRNA and protein α-SMA and collagen I.	[179]
	Simvastatin	25 mg/kg given 3 and 23 h after LPS challenge, or 25 mg/kg/day, from 3 days before LPS injection	Male Wistar rats administered with LPS and evaluated 6 and 24 h later	↓ Inflammation, leukocyte infiltration	↓ PP	↓ Sinusoidal endothelial dysfunction, ↑ eNOS phosphorylation	[180]
	Simvastatin	25 mg/kg/day in CCl_4_ and TAA-induced ACLD animals, 5 mg/kg/day in BDL-induced animals for 3 days and a last dose 30 min before the LPS injection	ACLD rats (CCl_4_, BDL or TAA) subjected to ACLF challenge with an injection of 1 mg/kg of LPS before the study	↓ ACLF-derived complications, ↑ survival, ↓ inflammation	↓ PP	↑ Sinusoidal function, ↓ LPS-mediated activation of HSC	[181]
	Simvastatin	5 mg/kg/day starting 3 days before the experiments	Male Sprague-Dawley cBDL-cirrhotic rats subject to hemorrhage/resuscitation	↓ ALT, AST, ↓ RNA expression of inflammatory genes	↓ Vasoconstriction	-	[182]
	Simvastatin	5 mg/kg/day for 15 days by gavage	Male Wistar rats (16 months old) with ACLD (CCl_4_).	↓ Fibrosis,	↓ Hepatic microvascular dysfunction,	↑ Fenestration, ↑ markers of hepatocyte function, ↓ markers of HSC activation	[183]
	INT-747 (FXR receptor agonist)	TAA-cirrhotic rats received two doses of 30 mg/kg 24 and 4 h before the experiments. Another grup of TAA and BDL-cirrhotic rats received 30 mg daily and 5 mg/kg every 2 days for 10 days, respectively.	Male Wistar TAA or cBDL-cirrhotic rats	-	↓ PP, ↓ HVR, ↑ hepatic microvascular function	↑ NO	[184]
	OCA (FXR agonist)	In vivo: 10 mg/kg either every 2 days during the last 4 weeks of the TAA intoxication or every 2 days for 4 weeks after cirrhosis development. In vitro: 0.1, 1 and 10 μM	In vivo: Male Wistar TAA-cirrhotic rats In vitro: hepatocytes, LSEC, HSC and Kupffer cells isolated from mice liver	In vivo: ↓ liver fibrosis, ↓inflammation	↓ PP, ↓ HVR	↓ LSEC activation, ↓ Kupffer cell activation, ↓HSC activation	[185]
Targeting other vascular alterations	Enoxaparin (anticoagulant)	In vivo: 1.8 mg/kg. -Short-term: Daily for 1 week (CCl_4_) -Long-term: Daily for 3 weeks (CCl_4_ and TAA) -Preventive: Daily for the last 3 weeks of the induction of cirrhosis	In vivo: Male Wistar CCl_4_ or male Sprague-Dawley TAA-cirrhotic rats In vitro: primary HSCs isolated from CCl_4_-cirrhotic rats	In vivo: ↓ liver fibrosis, ↑liver function, ↓ liver microthrombosis	↓ PP, ↓ HVR	In vivo: ↓ HSC activation, ↓ oxidative stress, In vitro: improved HSC phenotype	[186]
	Rivaroxaban (Anticoagulant)	In vivo: 20 mg/kg/day for 2 weeks. In vitro: 25, 50, 100 ng/mL during 24 h	In vivo: Wistar CCl_4_ or Sprague-Dawley TAA-cirrhotic rats In vitro: primary HSCs isolated from CCl_4_-cirrhotic rats	No regression of fibrosis	↓ PP	In vivo: ↑ NO (CCl_4_), ↑ LSEC phenotype, ↓HSC activation, ↓ liver microthrombosis. In vitro: ↑ HSC phenotype	[187]
	Sorafenib (multikinase inhibitor)	In vivo: 2 mg/kg/day for 2 weeks in PPVL rats. 1 mg/kg/day for 2 weeks in cBDL-cirrhotic rats.	Male Sprague-Dawley PPVL or cBDL-cirrhotic rats (PPVL, cBDL)	↓ splanchnic neovascularization, ↓inflammation, ↓ liver damage, ↓ liver fibrosis, ↓ angiogenesis	↓ PP (cBDL)	-	[188]
	Sorafenib, Imatinib or the combination of both (multikinase inhibitors)	30 mg/kg or 50 mg/kg, respectively, five times/week for 3 weeks	Female Balb/c Concanavalin A-acute liver fibrosis mice	↓ liver fibrosis	-	↓ HSC activation	[189]
Cell death and inflammation	Emricasan (Caspase inhibitor)	10 mg/kg/day for 7 days, starting 1 week after the animals developed cirrhosis	Male Wistar CCl_4_-cirrhotic rats	↓ AST, ↑ Bile, ↓ fibrosis, ↓ inflammation, ↑ hepatocyte phenotype	↓ PP, ↓ PPP, ↑ vasodilation	↓ Cell death, ↓ HSC activation and number, ↑ LSEC fenestrae, ↓ vWF, ↑ NO, improved HSC, LSEC and KC phenotype	[190]
	Emricasan	5, 25 or 50 mg daily for 24 weeks	196 patients with NASH cirrhosis	-	No significant differences in HVPG. Small ↓ HVPG in the compensated subgroup.	-	[191]
	Seolnsertib (ASK1 inhibitor)	18 mg or 6 mg daily for 48 weeks	Two phase III trials: 802 and 877 patients with NASH cirrhosis	No regression of fibrosis	-	-	[192]
	Fenofibrate (PPARα agonist)	25 mg/kg daily for 7 days	Male Wistar CCl_4_-cirrhotic rats	↓ liver fibrosis	↓ PP, ↑ MAP	↓ HSC activation, ↑ NO bioavailability, ↑ hepatic microvascular function	[193]
	Fenofibrate, Lanifibranor pioglitazone and GW501516 (PPAR agonists)	100, 30, 30, 10 mg/kg/day via oral gavage for up to 6 weeks	Choline-deficient, amino acid-defined high fat diet and WD-fed CCl_4_-cirrhotic rats	↓ liver fibrosis, ↓steatosis, ↓liver injury	-	↓ HSC activation	[194]
	Lanifibranor (pan-PPAR agonist)	In vivo: 100 mg/kg/day for two weeks In vitro: 1, 3 or 10 µM for 24 h	In vivo: Male Sprague-Dawley TAA or cBDL-cirrhotic rats In vitro: Primary human liver cells from patients with cirrhosis	In vivo: ↓ liver fibrosis, ↑ liver function, ↓ inflammation	↓ PP, ↓ HVR	In vivo: ↓ HSC activation, improved LSEC phenotype, ↑ hepatic microvascular function In vitro: improved HSC phenotype, ↓ HSC contraction capacity	[195]
	Liraglutide (GLP-1 analogue)	In vivo: 0.5 mg/kg/day for 15 days. In vitro: 50 µM for 72 h	In vivo: Male Wistar TAA-cirrhotic rats In vitro: Primary human HSC from patients with cirrhosis and Immortalized human-activated HSC LX-2	In vivo: ↓ liver fibrosis	↓ PP	In vivo: ↓ HSC activation, improved LSEC phenotype, ↑ hepatic microvascular function In vitro: ↓ HSC activation, ↓ HSC contraction capacity, ↓ inflammation	[196]
	Liraglutide	1.8 mg/day for 48 weeks	52 patients with NASH	↓ hepatic steatosis, ↓ hepatocyte ballooning	-	-	[197]
	Semaglutide (GLP-1 analogue)	0.4 mg/day for 72 weeks	162 patients with NASH	↑ NASH resolution. No improvement in fibrosis stage	-	-	[198]
	Metformin	300 mg/kg/day for 1 week	In vivo: Male Wistar CCl_4_ or Sprague-Dawley cBDL-cirrhotic rats In vitro: LX-2 cell line	In vivo: ↓ liver fibrosis, ↓ inflammation	↓ PP, ↓ HVR	In vivo: ↓ HSC activation, ↑ NO bioavailability, ↓ oxidative stress In vitro: ↓ markers of HSC activation	[199]
	ObR-Ab (Leptin receptor antagonist)	8 µg/kg/day for 1 week	Male Wistar CCl_4_-cirrhotic rats	-	↓ PP	↑ GMPc, ↓ oxidative stress	[200]
Lifestyle and dietary interventions	Docosahexaenoic acid	After cirrhosis induction, 500 mg/kg/day for two weeks	Male Sprague-Dawley TAA-cirrhotic rats	Recovery of normal fatty acid enzymatic activity and fatty acid composition, ↓ oxidative stress, ↓ inflammation, ↓ steatosis	↓ PP	↓ HSC activation, ↓ ECM accumulation	[201]
	VSL#3 (probiotics)	900 billion CFU per day for 6 weeks	17 patients with cirrhosis	↓ bacterial translocation, ↓ inflammation	↓ HVPG	-	[202]
	VSL#3	900 billion CFU per day for 24 weeks	130 patients with cirrhosis with a recent episode of hepatic encephalopathy	↓ CTP and MELD scores, ↓plasma proinflammatory markers (TNFα, IL1β and IL6)	-	-	[203]
	VSL#3	900 billion CFU per day for 2 months	94 patients with cirrhosis	↓ TNFα	↓ HVPG	-	[204]
	CECT7765 (Pseudocatenulatum)	1 billion CFU daily for 1 week	Male Sprague-Dawley cBDL-cirrhotic rats	↑ liver function: ↑ bilirubin and alkaline phosphatase. ↓ inflammation: ↓ TNFα, IL-6 and NO. ↑ FXR and eNOS gene expression. ↓ iNOS and COX-2	↓ portal vein area and portal flow		[205]
	VSL#3	900 billion CFU twice daily for 2 months	8 cirrhotic patients	-	No changes in HVPG	-	[206]
	VSL#3	900 billion CFU twice daily for 2 months	17 patients with decompensated cirrhosis and HVPG ≥10	-	No changes in HVPG	-	[207]
	Curcumin	50 mg/kg suspended in 0.5% carboxymethyl cellulose daily for 6 weeks	Male Sprague-Dawley rats with NASH induced by HFD	↓ inflammation, ↓ steatosis, ↓ insulin resistance		↓ MDA, ↑ hepatic GSH content, ↑ SOD activity, ↑ HO-1	[208]
	Resveratrol	10 and 20 mg/kg/day for 2 weeks	In vivo: Male Wistar CCl_4_-cirrhotic rats In vitro: LX-2 cell line	In vivo: ↓ liver fibrosis, ↓inflammation	↓ PP	In vivo: ↑ hepatic microvascular function, ↓ oxidative damage, ↓ HSC activation. In vitro: ↓ HSC activation markers	[209]
